# Directed Formation of DNA Nanoarrays through Orthogonal Self-Assembly

**DOI:** 10.3390/molecules16064912

**Published:** 2011-06-15

**Authors:** Jonathan R. Burns, Jurgita Zekonyte, Giuliano Siligardi, Rohanah Hussain, Eugen Stulz

**Affiliations:** 1 School of Chemistry, University of Southampton, Highfield, Southampton SO17 1BJ, UK; 2 National Centre for Advanced Tribology, School of Engineering Sciences, University of Southampton, Highfield, Southampton, SO17 1BJ, UK; 3 Diamond Light Source, Harwell Science and Innovation Campus, Didcot, Oxfordshire, OX11 0DE, UK

**Keywords:** terpy-DNA, AFM, TEM, DNA nanotubes

## Abstract

We describe the synthesis of terpyridine modified DNA strands which selectively form DNA nanotubes through orthogonal hydrogen bonding and metal complexation interactions. The short DNA strands are designed to self-assemble into long duplexes through a sticky-end approach. Addition of weakly binding metals such as Zn(II) and Ni(II) induces the formation of tubular arrays consisting of DNA bundles which are 50-200 nm wide and 2-50 nm high. TEM shows additional long distance ordering of the terpy-DNA complexes into fibers.

## 1. Introduction

**Figure 1:**
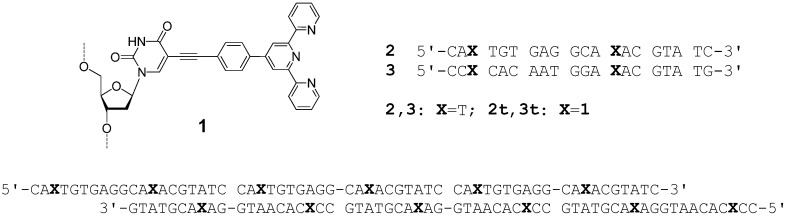
Structure of the terpyridine substituted thymidine monomer **1** as incorporated into the DNA strands, and sequences of the synthesised DNA strands. The assembly of the sticky ends of the DNA forms extended terpy-DNA strands as indicated below.

Since the seminal work of Seeman [1,2,3], Turberfield [4,5,6,7], LaBean [8,9], Rothemund [10,11], Yan [12,13], Mirkin [14,15] and others [16,17,18,19,20,21,22] in the field of DNA nanotechnology, the use of DNA to form novel self-assembled nanostructures has gained much attention with a view to creating functional entities for diagnostics, nanomachines or for use as biological templates. Self-assembled nano-structures, e.g., based on proteins and DNA, are widespread in Nature, where they play important roles in the structure and function of biological systems. To obtain a better understanding and to utilise these higher-order properties in the design of new functional materials based on biologically derived templates, we need reliable construction methods. In this respect, DNA is an ideal scaffold from which we can build supra-molecular structures, with high predictability, specificity and reliability [23,24,25,26,27]. The attachment of various functional groups to nucleosides and their subsequent sequence specific incorporation into DNA strands has reached a level of diversity where organic chemists can attach virtually any moiety imaginable. We can design DNA strands which contain modifications either at the end of the DNA [28,29], or within the DNA duplex itself. In the latter case, the modifications may be placed either on the outside of the DNA, *i.e.*, within the major or minor groove [30,31,32,33,34,35,36,37], or the nucleoside can be replaced with designer molecules to form interior stacked arrays of aromatic molecules or metal complexes [38,39,40,41]. The design strongly depends on the aim of the obtained material. Herein, we present the controlled formation of nanotubes starting from polymeric DNA duplexes containing a terpyridine deoxyuridine modification at specific positions, which are designed to be placed within the major groove of the B-type DNA and are accessible for metal complexation. The system makes use of orthogonal binding modes through selective metal recognition together with Watson-Crick base pairing of sticky ends of the DNA (Figure 1). Terpyridine or phenanthroline modified DNA have previously been shown to be useful in modulating DNA duplex stability through metal complexation between two complementary strands [42,43,44], or in the formation of specific supramolecular DNA structures [45,46]. A bisNTA-Ni(II)-His_6_ complex was recently used to create DNA arrays on surfaces [47]. In our system, the terpyridine is linked to the nucleoside *via* a short rigid linker that allows positioning of the metal complex at more precise orientation, in contrast to previously reported flexible linkers. The design of the DNA system is such that attachment of the terpyridine at every tenth position in the single strand will lead to a modification at every fifth position in the annealed duplex. Thus, the terpyridines will have an even spacing along the helical DNA at about half and full helical turns.

## 2. Results and Discussion

**Figure 2:**
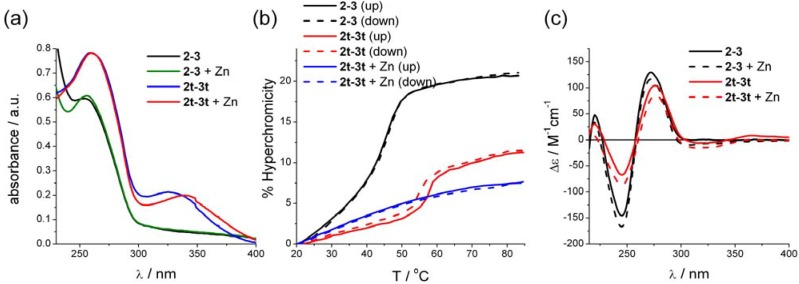
(**a**) UV-vis spectra of **2•3** and **2t•3t** in the presence and absence of Zn(II) (**b**) Thermal denaturing of the DNA strands **2•3**, **2t•3t** and of **2t•3t** in the presence of 0.5 eq Zn(II). (**c**) CD spectra of **2•3** and **2t•3t**, both in the presence and absence of Zn(II). Conditions for the measurements: 2 μM DNA in 100 mM NaCl, 50 mM phosphate buffer pH 7.0; the CD measurements were done at 4 μM concentration.

The synthesis of the terpyridine monomer **1** and its incorporation into DNA was performed using methods published previously [48]. The phosphoramidite of **1** couples efficiently during DNA synthesis using an increased coupling time of six minutes, and yields are in the range of 50-70% on a 1 μmol scale. The DNA strands were purified using fluorous tag affinity chromatography [49,50] and characterised using HPLC, UV-vis and circular dichroism (CD) spectroscopy. The terpy containing DNA strands **2t** and **3t** show the characteristic terpy absorbance with *λ*_max_ = 325 nm (Figure 2a), confirming the incorporation of the terpy monomer unit; the absorbance of **1** does not change upon duplex formation. 

The thermal denaturing of the unmodified DNA duplex **2•3** shows a relatively clear transition with a *T*_m_ = 45.4 °C; however, the curvature of the lower temperature melting profile suggests cooperative denaturing because of the sticky end interactions (Figure 2b). Surprisingly, the terpy modifications have a significant stabilising effect on the DNA duplex, where **2t•3t** shows a *T*_m_ = 56.3 °C. This *T*_m_ is also closer to the *T*_m_ calculated for the fully matched sequence (61.3 °C). Here, the cooperativity is even more pronounced, and a hysteresis of 2 °C indicates different kinetics upon melting and annealing of the strands. Also, the hyperchromicity is lower than for **2•3**. The nature of the stabilising effect of the tepy unit is unclear at this point. The CD spectra of both natural and modified DNA are very similar in shape (Figure 2c), thus it can be assumed that both systems adopt a B-type DNA structure at ambient temperature [42]. The terpy unit itself gives rise only to a very weak and broad induced signal. The intensities of the terpy-DNA **2t•3t** peaks are lower than those of the unmodified DNA, in particular for the peak at *λ* = 244 nm, thus the terpy unit does seem to have some effect on the local structure of the DNA.

If 0.5 equivalents of ZnCl_2_ per terpy are added, the melting behaviour of the terpy-DNA **2t•3t** changes markedly (Figure 2b), but not that of the unmodified duplex **2•3** (not shown). The melting curves of **2t•3t** lose the pronounced features of melting transitions, and only a steady increase in absorbance is observed. This is highly indicative of formation of intermolecular terpy-metal complexes, which induce formation of larger arrays through non-covalent connection of the DNA strands, and is likely to lead to a highly cooperative denaturing process which would explain the lack of clear melting transitions. The UV-vis spectrum of **2t•3t** shows a distinctive change in the terpy absorbance region with a bathochromic shift of 24 nm to *λ*_max_ = 339 nm. The change in the CD spectrum is such that the signal is almost completely suppressed. Therefore, it can be assumed that all terpy units are complexes with Zn(II). Interestingly, both duplexes **2•3** and **2t•3t** show similar changes in the DNA region of the CD spectrum. The addition of metal reduces the peak at 275 nm by 10% for **2•3** and 18% for **2t•3t**, whereas the peak at 245 nm increases by 15% for **2•3** and 33% for **2t•3t**. Thus the addition of metal ions does affect the overall structure of the duplexes, where the effect is much more pronounced for the terpy-DNA.

**Figure 3:**
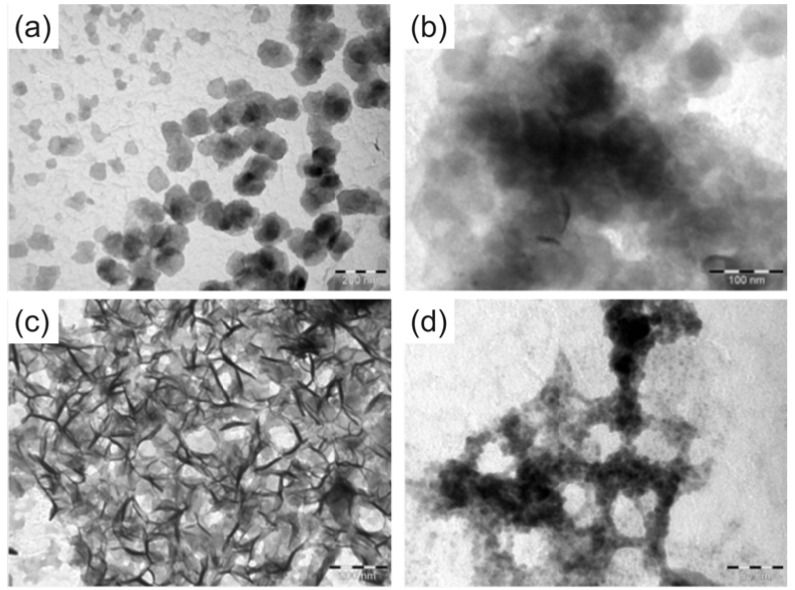
TEM images of the DNA arrays obtained after annealing and staining, in the presence or absence of Zn(II). (**a**) **2•3** (scale bar: 200 nm). (**b**) **2t•3t** (scale bar: 100 nm). (**c**) **2t•3t** + Zn(II) (scale bar: 200 nm). (**d**) **2t•3t** + Zn(II) zoomed in (scale bar: 50 nm).

To obtain an idea about possible arrays being formed through metal complexation, we analysed the global structure using Transmission Electron Microscopy (TEM) and Atomic Force Microscopy (AFM). The samples were slowly annealed overnight in the presence or absence of metal ions. For the TEM analyses, the samples were dried on a copper grid and stained using uranyl acetate and lead citrate. Both **2•3** and **2t•3t** display relatively unordered organic matter (Figure 3); this does not change for **2•3** upon addition of Zn(II). Thus the interactions as seen in the CD spectra do not have a long range effect on the structure. On the other hand, the terpy-DNA duplexes **2t•3t** exhibit a very different picture. The complexation of the terpy groups with the metal ions leads to a long range ordering of the DNA into fibre like structures. Higher magnification of **2t•3t**-Zn shows that the fibres actually seem to consist of arrays of small globular structures of an approximate size of 2 to 5 nm.

**Figure 4:**
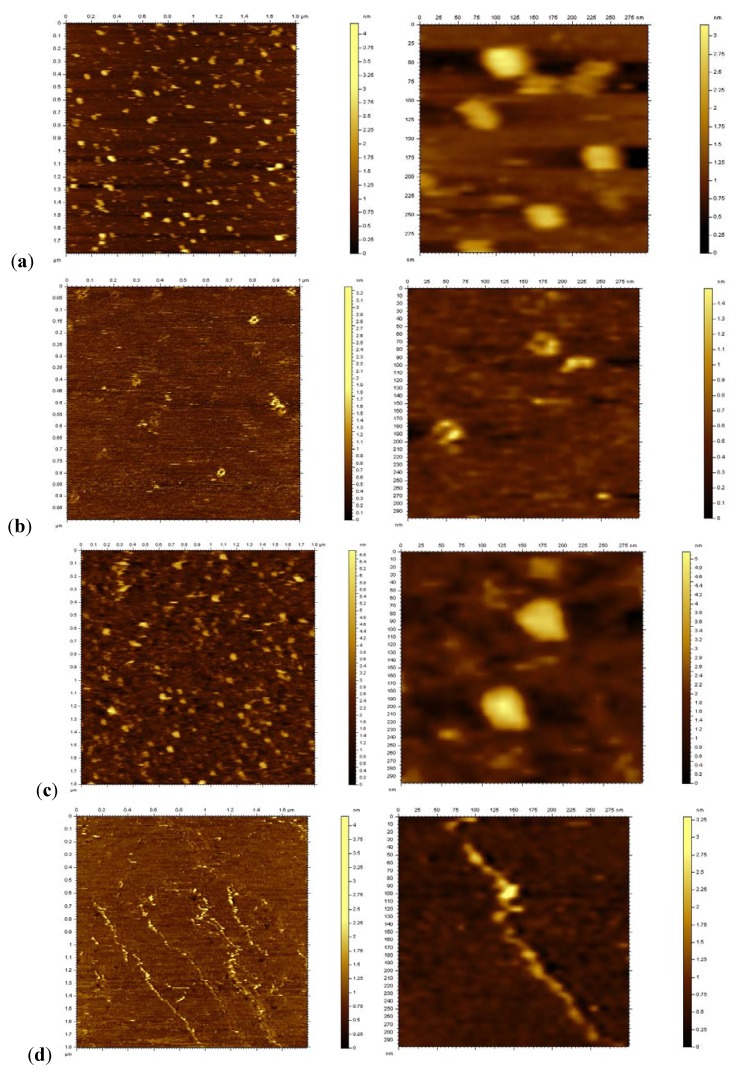
AFM images of the DNA systems. (**a**) **2•3**. (**b**) **2•3** + Zn(II). (**c**) **2t•3t**. (**d**) **2t•3t** + Zn(II). Scan size is 1.8 μm (left) and 300 nm (right) for all pictures except (**b**), which is 2 μm and 300 nm.

To obtain a more detailed picture we probed the structures by AFM (Figure 4). The samples were measured under aqueous conditions to maintain a more natural environment for the DNA. After depositing the samples on a freshly cleaved mica surface, the DNA was left for two hours to settle. In contrast to what we would have expected, but in-line with the TEM pictures, both DNA systems do not display long strands through annealing of the sticky ends. Rather the DNA forms globular assemblies of variable size, which range from about 10 to 100 nm. This indicates that the sticky ends are not stable enough to assemble a larger amount of DNA units. Surprisingly, the addition of Zn(II) to the duplex **2•3** disrupts the globular assemblies and the duplexes rearrange into small circular structures of 400-500 nm diameter. The driving force for this transformation is not quite clear at this point, but most likely has to do with interactions between the metal ions and the phosphate backbone of the DNA. The terpy-DNA **2t•3t** again behaves very differently: it might have been expected that the complexation with the metal ions will connect multiple DNA strands to form flat ordered layers of DNA, similarly to what was reported previously with Ni(II)-DNA complexes [47]. The AFM pictures reveal that the Zn(II)-terpy-DNA forms tubular structures of 2-5 μM length. The width and height of those assemblies varies between 100-300 nm and 30-50 nm, respectively. A computer model of **2t•3t** shows that the terpy units, when looking down the helical axes of the DNA, are not aligned at 180°; the angle formed is actually 135°. 

Also, when considering long DNA strands the terpyridines are not exactly at full helical turns which would be 10.5 base pairs in B-DNA; some flexibility in the helicity of the DNA might, however, compensate for this slight out-of-phase arrangement. The formation of interstrand cross-links therefore leads to a system which forms tubular structures as thermodynamically most stable systems, similar to multi-walled nanotubes, rather than planar arrays of linearised DNA strands.

## 3. Experimental

*General*

The synthesis of the terpyridine-dU building block **1** and its incorporation into the DNA strands **2t** and **3t** was performed as described earlier [48]. UV-Vis analyses were performed using a Varian Cary 300 Bio UV-vis spectrophotometer in 1 cm quartz cells. Concentrations were made to 2 μM in 0.1 M sodium phosphate buffer (pH 7.0) using an ε(260)-value of 195700 M^−1^ cm^−1^ for **2** and 196200 M^−1^ cm^−1^ for **3**. The DNA was annealed and denatured at 1 °C / min using the Varian peltier device. Circular Dichroism spectra were collected using B23 beamline at Diamond Light Source using Module B end-station with 0.5 mm slit equivalent to 1.2 nm bandwidth, 1 s integration time, 38 nm/min scan speed at a wavelength range 190-400 nm. Solutions were made to 4 μM in 0.1 M sodium phosphate buffer (pH 7.0). TEM analysis was performed using a Hitachi H7000 TEM, operating at a bias voltage of 75 kV. 10 μL of the 2 μM annealed DNA was loaded on to carbon film 400 mesh copper (50) TEM grids (Agar Scientific), left to dry and stained using lead citrate stain [51] and uranyl acetate 2% aqueous solution. AFM topographical images were acquired in buffer at room temperature with magnetically driven dynamic force microscopy (MAC Mode III, 5500 Scanning Probe Microscopy from Agilent Technologies, US), and magnetically coated Type VI MAC levers (Agilent Technologies, US). The nominal spring constant of MAC lever was 0.2 N/m, resonance frequency 22 kHz, and the lateral scan frequency was 2 Hz. PicoView 1.6 and PicoImage (Agilent Technologies, US) software were used for data acquisition and image analysis, respectively. For AFM imaging, DNA samples were deposited onto freshly cleaved mica substrate attached to the commercial fluid cell. The mica discs (14 mm, Agar Scientific) were pre-treated with NiCl_2_ solution according to established methods [52] to which the DNA arrays were loaded; AFM imaging was performed after 1h deposition time.

## 4. Conclusions

**Figure 5:**
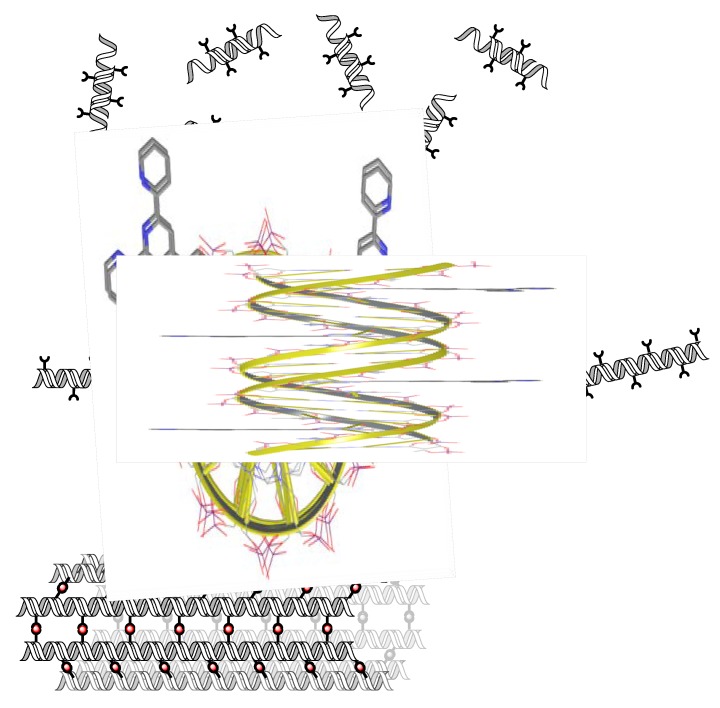
Putative representation of the annealing of long DNA strands through sticky-end recognition and formation of tubular assemblies by metal complexation.

We have shown that terpyridine nucleosides, where the terpy unit is attached through a rigid linker, can be addressed selectively with metals and be used to form non-covalent interstrand DNA linkages. The DNA strands form, after addition of metal ions, long tubular assemblies rather than planar arrays. Because the terpy is not flexible in its position, this seems to be guiding the 3D structure towards the observed linear structures and represent the thermodynamically most favourable arrangement, which is obtained after slow annealing (Figure 5). 

The short and rigid linker is also expected to prevent intercalation, which might otherwise contribute to the formation of superstructures. Our system is therefore orthogonal to other reported metal complex-substituted DNAs. It should be noted that the same results are obtained when the metal is added after or during annealing of the DNA, and also when zinc is replaced with nickel (not shown); this did not alter the spectroscopic properties of the DNA. The process of assembly can therefore be either step-wise or concerted; given the reversibility of the interactions (hydrogen bonding, metal complexation) a concerted mechanism would seem more likely. We are now probing different sequences and metal ions to obtain a wider range of arrays through modulation of orientation and stability of the metal complexes.

## Supplementary Materials

Supplementary File 1
