# Supplementary material for: Directed Formation of DNA Nanoarrays through Orthogonal Self-Assembly
Source: Molecules. 2011 Jun 15;16(6):4912–22. doi: 10.3390/molecules16064912 (PMC6264196; doi:10.3390/molecules16064912)

Molecules **2011** *16*

OPEN ACCESS
molecules
ISSN 1420-3049
www.mdpi.com/journal/molecules

Supporting Information

Directed formation of DNA Nanoarrays through orthogonal self-assembly

Jonathan R. Burns, Jurgita Zekonyte, Giuliano Siligardi, Rohanah Hussain, and Eugen Stulz

TEM pictures of DNA:

Control system **2•3** (left), in the presence of Zn(II) (middle) and Ni(II) (right).

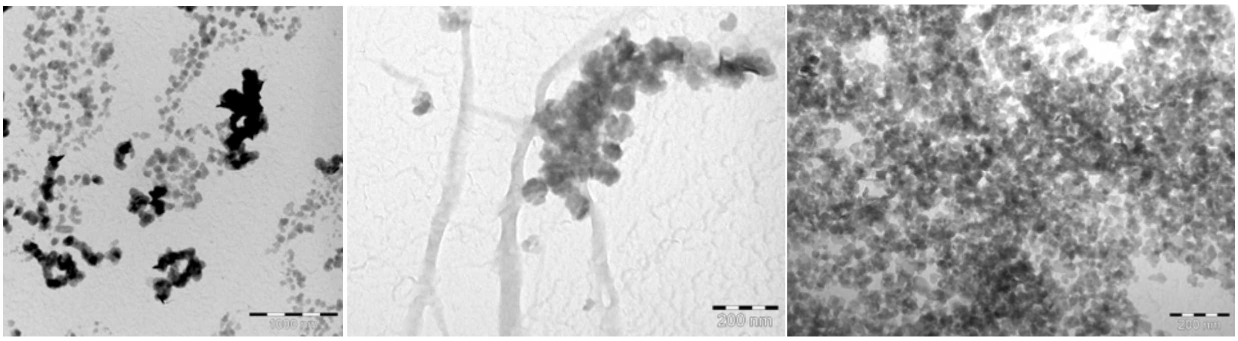

Terpyridine system **2t•3t** in the absence of Zn(II)

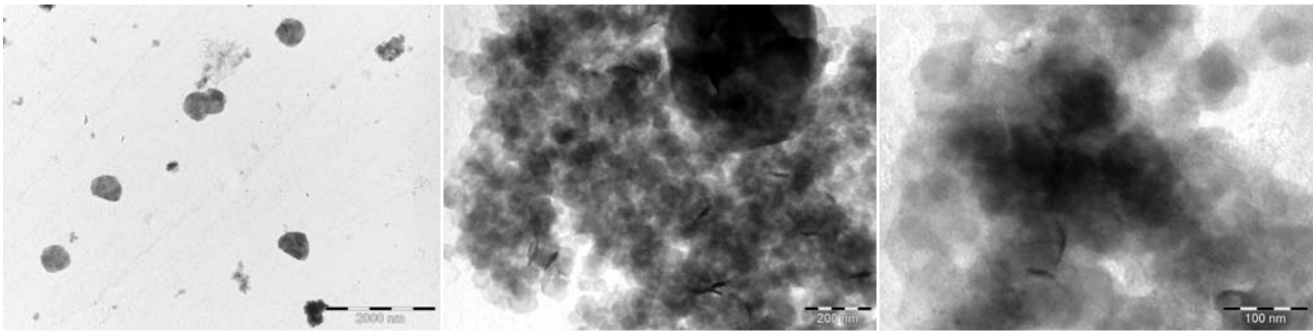

Terpyridine system **2t•3t** in the presence of Zn(II) (left, middle) and Ni(II) (right)

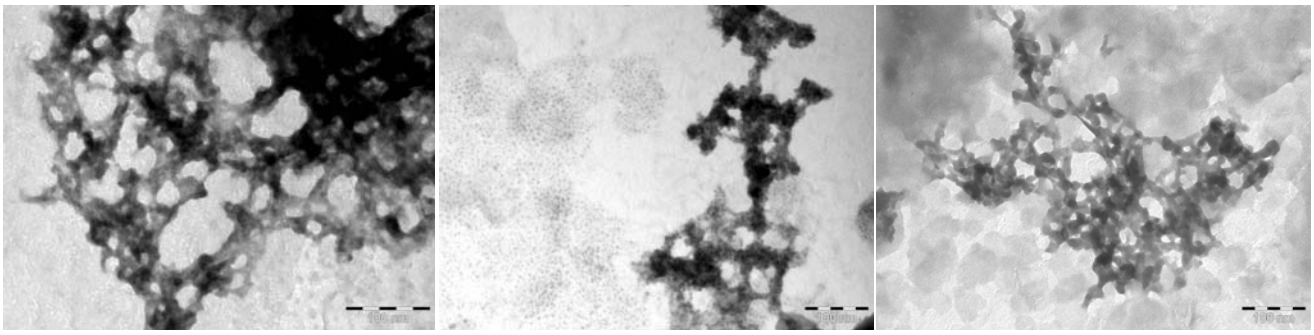

AFM pictures of DNA:

Control system **2•3** in the absence of Zn(II)

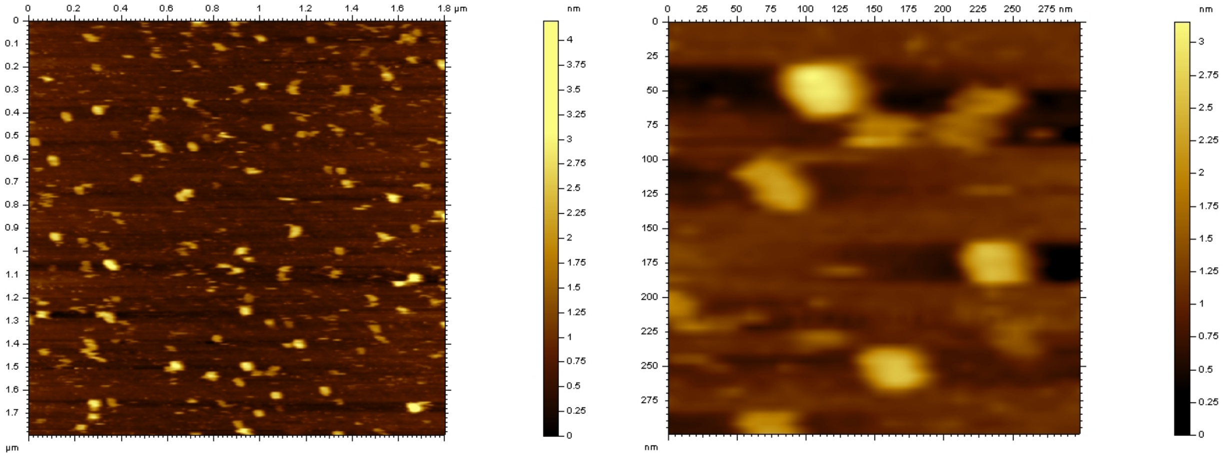

Control system **2•3** in the presence of Zn(II)

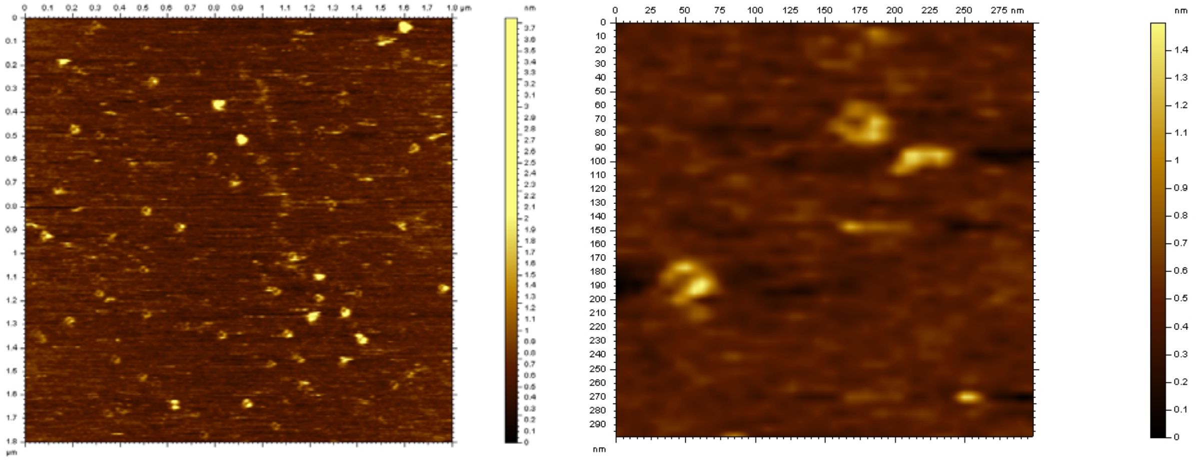

Terpyridine system **2t•3t** in the absence of Zn(II)

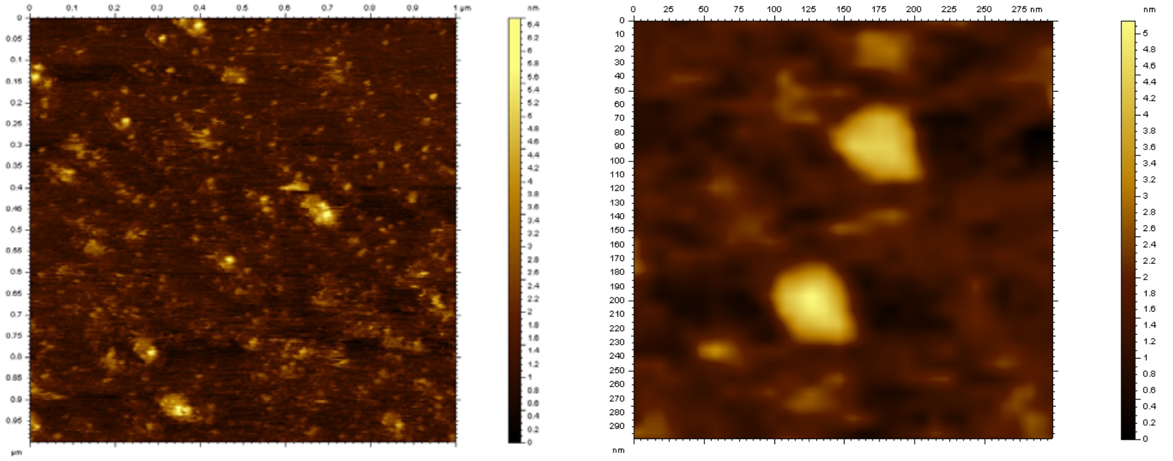

Terpyridine system **2t•3t** in the presence of Zn(II)

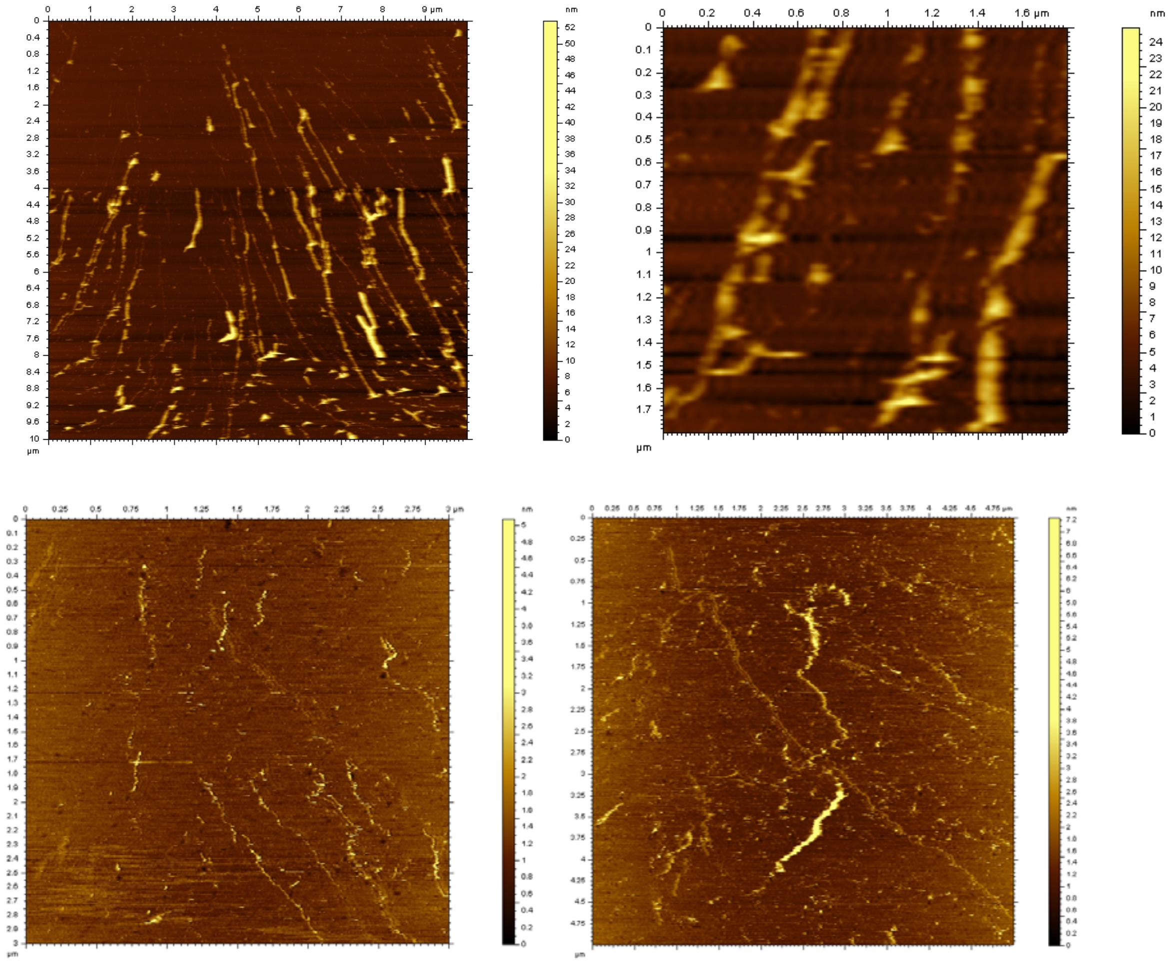

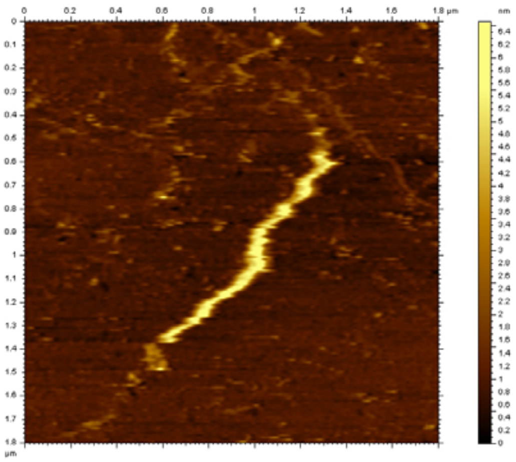

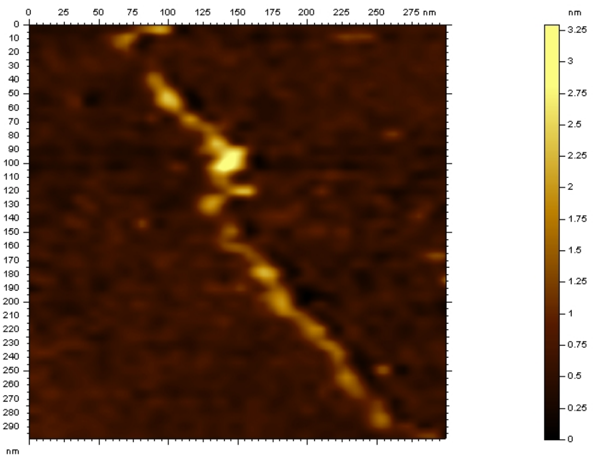

Supplement: Supplementary File 1 [file molecules-16-04912-s001.pdf]
